# Dental pain among children aged 8 to 11 and associated factors: a population-based study

**DOI:** 10.1590/1807-3107bor-2025.vol39.120

**Published:** 2025-11-17

**Authors:** Taiane Oliveira SOUZA, Luana Leal ROBERTO, Nathalia Geovana Corrêa RUAS, Débora SOUTO-SOUZA, Paula Cristina Pelli PAIVA, Maria Letícia RAMOS-JORGE

**Affiliations:** (a) Universidade Federal dos Vales do Jequitinhonha e Mucuri – UFVJM, School of Dentistry, Department of Dentistry. Diamantina, MG, Brazil.; (b) Faculdade de Ciências e Tecnologia de Janaúba – Facitec, School of Dentistry, Janaúba, MG, Brazil.; (c) Faculdades Unidas do Norte de Minas – Funorte, School of Dentistry, Department of Dentistry, Montes Claros, MG, Brazil.; (d) Centro Universitário do Triângulo – Unitri, School of Dentistry, Department of Dentistry, Uberlândia, MG, Brazil.

**Keywords:** Child, Toothache, Pediatric Dentistry, Epidemiology

## Abstract

This study aimed to identify factors associated with dental pain among schoolchildren in Diamantina, Minas Gerais, Brazil. A total of 627 children aged 8–11 years were assessed through questionnaires and clinical examinations in both public and private schools. We employed a complex, probabilistic, two-stage cluster sampling method involving schools and classes. The sample size was calculated assuming a 50% prevalence of events or diseases, a 95% confidence level, a 5.0% margin of error, a design effect (deff) of 1.5, and a 15% non-response rate. The dependent variable, dental pain, was assessed by asking: “Have you ever had a toothache in your life?” with possible responses of “No” or “Yes.” The independent variables included sociodemographic factors and dental history. Binary logistic regression was used to estimate the odds ratio (OR). Approximately 70.6% of the children reported having experienced dental pain at least once in their lives. In the final model, the likelihood of having experienced dental pain was higher among children who had either deciduous or permanent teeth restored (OR = 1.99; 95%IC: 1.19–3.29), who had a normative need for dental treatment (OR = 3.00; 95%IC: 1.96–4.58), and whose guardians perceived their oral health negatively (OR = 1.81; 95%IC: 1.19–2.75). Dental pain is a significant oral health issue among children. This pain was associated with both unfavorable normative assessments and subjective perceptions of oral health, underscoring the importance of preventive and promotive strategies for children’s oral health.

## Introduction

Pain can be defined as a subjective experience associated with actual or potential tissue damage, characterized by both aspects. Pain perception is a multidimensional and complex experience that varies in quality and sensory intensity. Odontalgia is the most common^1^ type of orofacial pain.

Knowledge, beliefs, psychological state, and the social and cultural environment can influence both the presence and perception of pain. Factors such as less affluent economic conditions, dental caries, sleep disorders, and difficulty eating are associated with this perception.^2^

A child’s first visit to the dentist is an important event. Several changes in the oral cavity can affect children in early childhood, and professionals must be equipped to manage these changes. However, studies indicate that few children visit the dentist before the age of one, which often leads to caries and subsequent complications.^3^ Emergency care typically addresses severe pulp pain, infections, and dental trauma—conditions that can cause pain during dental appointments. Children who have never received dental treatment or who are unfamiliar with the dental setting and dentist may exhibit withdrawn behavior associated with pain, fear, and dental anxiety, potentially impacting them into adulthood.^4-6^

In Brazil, the oral health conditions of preschoolers are concerning. Despite recent epidemiological surveys showing improvements, such as a 6% increase in caries-free five-year-olds, this group still suffers from a high prevalence of oral health issues, including dental caries and malocclusion.^7^ These issues often lead to significant dental pain and, consequently, negatively affect their daily lives. However, few population-based studies with representative samples of Brazilian schoolchildren concerning dental pain^2^ have been identified. Therefore, this study adds to the existing literature by exploring the characteristics of the child population associated with dental pain.

Considering the significant impact of pain on children’s quality of life and their physical and mental well-being, this study aimed to investigate the factors associated with dental pain among children aged 8–11 in Diamantina, Minas Gerais, Brazil. It is hypothesized that dental pain in children is associated with unfavorable sociodemographic and health conditions.

## Methods

This cross-sectional study was conducted with schoolchildren aged 8-11 enrolled in public and private schools in the urban area of Diamantina, Minas Gerais (MG), Brazil, a municipality in southeastern Brazil with approximately 45,880 inhabitants.^8^

### Sampling

The study employed a complex, probabilistic, two-stage cluster sampling method (schools and classes). We calculated the sample size needed to estimate prevalence in a representative sample of the population. The calculations indicated a need for 692 individuals for the population aged 8–11, based on a 50% occurrence of events or diseases, a 95% confidence level, a 5.0% margin of error, a design effect (*deff*) of 1.5, and a 15% non-response rate. As the non-response rate was included in the sample calculation, a loss of up to 15% does not compromise the study results. Primary sampling units were selected by simple random selection, with schools chosen by lottery. In the second stage, classes were randomly selected from each participating school.

Children with the cognitive ability to read and write were eligible for this study. Exclusion criteria included children with systemic alterations reported by a doctor and those wearing orthodontic braces.

### Calibration

Data collection involved a note-taker and a dentist, trained by a professor of pediatric dentistry with extensive experience in diagnosing carious lesions at various stages and classifying dental trauma. Calibration was performed by evaluating diagnostic images on two separate occasions, one week apart, to assess intra-examiner reliability. The Kappa values obtained were above 0.80 for dental caries and above 0.85 for dental trauma, indicating significant agreement. The diagnostic criteria from the 4^th^ edition of the Oral Health Surveys: Basic Methods of the World Health Organization (WHO)^9^ were adopted.

### Data collection

The questionnaires and clinical examinations were conducted in a designated area within the children’s schools at a prearranged time and date between October 2017 and May 2018. For the clinical examinations, students were seated directly across from the examiner. Teeth were cleaned, dried with gauze, and examined using sterilized clinical instruments. Both natural and artificial lighting were utilized (DP. Led Light DP-722 A, Guangzhou, China).

Dental caries were assessed using the DMFT Index, as proposed by Klein and Palmer in 1937.^10^ For each individual examined, the DMFT index represents the total number of decayed, missing, and filled permanent teeth, with values ranging from zero to 32^11^ in the permanent dentition. The dmf-t index was used for the deciduous dentition, accounting for decayed teeth, teeth indicated for extraction, and filled teeth. The “extracted” condition is not included in this index, as it is clinically impossible to determine whether a lost tooth was due to decay or natural exfoliation in preparation for the emergence of a permanent tooth.^12^

Dental trauma was classified according to the O’Brien index,^13^ which records various types of dental injuries, including enamel fractures, enamel, and dentin fractures, crown fractures with pulp involvement, extrusive dislocation, lateral dislocation, intrusive dislocation, avulsion, discoloration, fistulae, and loss or restoration of teeth due to trauma.

The questionnaires were administered in a reserved area at a prearranged time and date within the children’s schools. Students began by filling out the Dental Pain questionnaire developed by Góes in 2001.^14^ The researcher read the questionnaires aloud, allowing all students to complete them simultaneously at the end of the reading. Once completed, the researcher collected the instruments, and each student retained their respective, numbered epidemiological clinical record. Clinical and epidemiological data were collected using these research instruments. Examinations were performed under natural light with a previously sterilized mirror and probe suitable for evaluating the Community Periodontal Index (CPI), using all codes and criteria proposed by the WHO.^9^ A self-administered Sociodemographic Questionnaire was used for guardians, which included questions on socioeconomic characteristics, use of dental services, and perceptions of oral health.

### Variables evaluated

The dependent variable, Dental Pain, was assessed by asking the question: “Have you ever had a toothache in your life?” with possible responses of “No” or “Yes.” The independent variables included were age group (8–9 years; 10–11 years), sex (female; male), type of school (private; public), maternal education (over 9 years of schooling; up to 9 years of schooling), household income (≥ 2 minimum wages; < 2 minimum wages), number of people living on household income (≤ 3 people; > 3 people), and type of housing (owned; not owned). Additionally, dental variables were considered: previous use of dental services (yes; no), time since last dental visit (less than 1 year; more than 1 year; never), frequency of daily brushing (three times; twice; does not brush every day), untreated caries in deciduous or permanent teeth (absent; present), restored deciduous or permanent teeth (absent; present), plaque on deciduous or permanent teeth (no; yes), bleeding in deciduous or permanent teeth (no; yes), dental trauma (absent; present), normative need for dental treatment (absent; present), and the guardian’s perception of the child’s oral health (excellent/good; fair/poor).

### Data analysis

Analyses were conducted using the Predictive Analytics Software (PASW - SPSS®, Chicago, EUA) version 20.0. All variables were categorical. Descriptive analysis provided absolute (n) and relative (%) frequencies. Bivariate analyses were conducted using Pearson’s chi-squared test. Variables with a p-value ≤ 0.2 were included in the multiple model. This model was adjusted using Binary Logistic Regression to estimate the Odds Ratio (OR) and 95% confidence interval (95% CI). Only variables with a significance level ≤ 0.05 were retained in the final model. The Hosmer-Lemeshow test was also performed to assess the model’s fit.

### Ethical issues

This study adhered to the ethical principles outlined in the National Health Council Resolution (CNS) N° 466/12 and was approved by the Human Research Ethics Committee of the Federal University of Jequitinhonha and Mucuri Valleys (Opinion N° 2.667.343). Informed consent was obtained from all participating parents/legal guardians. The children also agreed to participate by signing an Informed Assent Form.

## Results

In total, 627 of the 692 individuals estimated for the sample calculation were evaluated, representing a 9% loss. Of these, 438 (70.6%) reported having experienced dental pain at least once. The descriptive analysis indicated that most children were female (53.0%) and attended public schools (89.6%). Most children lived in households with a monthly income of less than two minimum wages, with 84.7% of these households comprising three or fewer people. Over half of the mothers had less than nine years of schooling. Regarding dental factors, 24.8% of the schoolchildren had never visited a dentist, 45.9% had untreated caries in deciduous or permanent teeth, and 57.3% were identified as having some need for dental treatment (Table 1).

**Table 1:** Descriptive and bivariate analysis of schoolchildren aged 08-11 years regarding dental pain. Diamantina (MG), 2018. (n = 627)

Variables	n	%	Dental pain				p-value
			Absent		Present		
			n	%	n	%	
Sociodemographic							
Age group (in years) *							0.254
8–9	372	60.8	116	31.3	256	68.7	
10–11	240	39.2	64	26.9	176	73.1	
Sex							0.158
Woman	332	53.0	89	26.9	243	73.1	
Man	295	47.0	95	32.1	200	67.9	
School type							< 0.001
Private	82	13.1	40	48.8	42	51.2	
Public	545	86.9	144	26.4	401	73.6	
Maternal schooling (study years)*							0.107
> 9	232	41.5	78	33.6	154	66.4	
≤ 9	327	58.5	89	27.2	238	72.8	
Household income* (minimum wages)							0.060
≥ 2	185	32.0	61	33.2	124	66.8	
< 2	393	68.0	101	25.6	292	74.4	
Number of people living on household income*							0.906
≤ 3	508	84.7	146	28.7	362	71.3	
> 3	92	15.3	27	29.3	65	70.7	
Housing type*							0.323
Owned	388	63.7	119	30.7	269	69.3	
Not owned	218	36.3	64	26.9	154	73.1	
Odontological variables							
Previous use of dental services*							0.407
Yes	458	75.2	129	28.2	329	71.8	
No	151	24.8	48	31.8	103	68.2	
Time since last visit* (year)							0.184
< 1	252	41.4	78	30.8	174	69.2	
> 1	206	33.8	51	24.9	155	75.1	
Never went to the dentist	151	24.8	47	31.8	101	68.2	
Daily brushing frequency* (times)							0.417
3	388	64.1	117	30.2	271	69.8	
2	182	30.1	51	27.9	131	72.1	
Does not brush every day	35	5.8	7	20.0	28	80.0	
Untreated caries in deciduous and/or permanent teeth*							< 0.001
Absent	335	54.1	133	39.8	202	60.2	
Present	284	45.9	49	17.3	235	82.7	
Restored deciduous and/or permanent teeth*							< 0.001
Absent	449	72.4	154	34.3	295	65.7	
Present	171	27.6	28	16.5	143	83.5	
Plaque on deciduous and/or permanent teeth*							0.115
No	25	4.2	11	44.0	14	56.0	
Yes	572	95.8	167	29.2	405	70.8	
Bleeding in deciduous and/or permanent teeth*							0.005
No	113	19.0	46	40.7	67	59.3	
Yes	483	81.0	131	27.2	351	72.8	
Dental trauma*							0.621
Absent	537	86.9	160	29.9	376	70.1	
Present	81	13.1	22	27.2	59	72.8	
Normative need for dental treatment*							< 0.001
Absent	234	42.7	104	44.6	130	55.4	
Present	314	57.3	54	17.2	260	82.8	
Parents’ perception of their child’s oral health*							< 0.001
Excellent/good	246	40.5	99	40.1	147	59.9	
Fair/poor	362	59.5	80	22.0	282	78.0	

*Total n variation due to loss of information.

The bivariate analysis revealed that dental pain was associated (p ≤ 0.20) with several variables, including sex (p = 0.158), type of school (p<0.001), maternal schooling (p = 0.107), and household income (p = 0.060). Dental variables such as time since the last dental visit (p = 0.184), untreated caries in deciduous or permanent teeth (p < 0.001), restored deciduous or permanent teeth (p < 0.001), plaque (p = 0.115), and bleeding in deciduous or permanent teeth (p = 0.005) also showed associations, as did the normative need for dental treatment (p < 0.001) and the guardian’s perception of their child’s oral health (p < 0.001) (Table 1).

In the adjusted multiple analysis, the likelihood of having experienced dental pain was higher among children with restored deciduous or permanent teeth (OR =1 .99; 95%IC: 1.19–3.29), those with a normative need for dental treatment (OR = 3.00; 95%IC: 1.96–4.58), and those whose guardians perceived their oral health negatively (fair/poor) (OR = 1.81; 95%IC: 1.19–2.75) (Table 2). The Hosmer-Lemeshow chi-square test yielded a value of 7.609 (p = 0.179).

**Table 2:** Binary logistic regression analysis of dental pain of schoolchildren aged 08-11 years. Diamantina (MG), 2018. (n = 530)

Odontological variables	OR (95%CI)	p-value
Restored deciduous and/or permanent teeth		
Absent	Ref.	0.008
Present	1.99 (1.19–3.29)	
Normative need for dental treatment		
Absent	Ref.	< 0.001
Present	3.00 (1.96–4.58)	
Parents’ perception of their child’s oral health		
Excellent/good	Ref.	0.005
Fair/poor	1.81 (1.19–2.75)	

OR: Odds ratio; Ref.: Reference category. X^2^ Hosmer and Lemeshow = 7.609 (p-value = 0.179).

## Discussion

This study revealed a higher likelihood of dental pain among children with restored deciduous or permanent teeth, those with a normative need for dental treatment, and those whose guardians perceived their oral health negatively. A notably high prevalence of dental pain (70.6%) was observed among the schoolchildren evaluated. In comparison, lower prevalence levels of dental pain were reported in national studies among 8- and 9-year-old schoolchildren in Belo Horizonte, Minas Gerais (45.9%),^15^ Brazil, and in international studies conducted with 8-year-old children in Sri Lanka (49%)^16^ and England (47.5%).^17^ It is important to note that the methods used to verify the prevalence of pain varied between this study and the referenced studies,^15-17^ where pain was assessed over different time frames: recent pain (last month),^15^ pain in the last two months^16^ and pain at any point in life. This variation in methodology may affect the comparability of the results. The high prevalence of dental pain identified in this study underscores the need for health promotion actions specifically targeting the child population to prevent the onset of dental issues. This is particularly crucial for children who have never received dental treatment or have not been exposed to a dental environment, as they may exhibit withdrawn behavior associated with pain, fear, and dental anxiety, potentially impacting their well-being into adulthood.^4-6^

Children with restored deciduous and permanent teeth were more likely to have experienced dental pain at some point. Although this is a cross-sectional study, and it is impossible to establish causality from the associations observed, it is possible that the children had experienced pain before their teeth were restored. This association underscores the role of pain in prompting children to seek dental care. A previous study reported that caries and its complications were the most common reasons for children’s initial dental visits.^3^ Another study indicated that most parents arranged dental appointments for curative reasons (such as caries, endodontic treatments, delayed eruption, and pain), while only a small fraction (22%) arranged preventive appointments.^18^

The final model showed that children in need of dental treatment were more likely to report dental pain than those without such needs. Supporting this, a prior study found that children requiring dental treatment for three or more teeth were approximately four times more likely to experience recent dental pain compared to those without treatment needs,^19^ which aligns with the findings of this study.

The guardian’s perception of their child’s oral health was independently associated with dental pain, with children more likely to experience dental pain when their guardians perceived their oral health as fair or poor. Studies have shown that parents of children with dental caries and dental pain often rate their child’s oral health as fair or poor.^20^ A study among Brazilian children aged 3-5 years revealed that guardians only considered the child’s oral health poor when dental caries were accompanied by dental pain.^21^ This suggests that guardians may delay seeking dental care until significant oral health issues arise, as few children visit a dentist before the age of one, which can lead to caries and its painful complications.^3^ Given these findings, health promotion, prevention, and access to dental services are vital to minimizing dental pain among children.

Dental pain remains a significant health issue globally, making it crucial to understand its associated factors to mitigate new episodes.^22^ The results of this study underscore the importance of both preventive and curative dental approaches in clinical practice and public health initiatives. In the clinical setting, the findings reinforce the need for ongoing education for those responsible for maintaining oral health. Moreover, parents’ negative perceptions of their children’s oral health could influence their willingness to seek and adhere to dental treatment,^23^ necessitating improved communication between professionals and families. The results also emphasize the importance of thorough clinical monitoring for children at risk of developing dental caries and those with restored teeth to reduce the incidence of dental pain.

In public health, the high prevalence of dental pain underscores the urgency for more comprehensive preventive programs and increased access to preventive dental treatments to reduce the need for restorative interventions, which are often associated with a greater likelihood of experiencing pain. Implementing these measures is crucial to mitigating childhood oral health issues and enhancing children’s quality of life.

One limitation of this study is its cross-sectional design, which inherently imposes temporal limitations. Additionally, only quantitative methods were used in this investigation, whereas a combination of quantitative and qualitative methods is often recommended for exploring subjective health outcomes. Importantly, the age range used in this study (8 to 11 years) does not align with the index ages and age ranges recommended by the WHO for measuring oral health problems,^9^ which may limit the ability to draw parallels and make comparisons. Therefore, future studies should consider using both quantitative and qualitative methods and adopt the index ages and age ranges recommended by the WHO^9^. On the other hand, the robust sampling plan, thorough examiner calibration, and multiple analyses have ensured the validity and reliability of the data, making the results representative of the target population.

## Conclusion

Dental pain among children aged 8 to 11 was associated with restored deciduous or permanent teeth, a normative need for dental treatment, and guardians’ negative perceptions (fair/poor) of oral health. Given these findings, it is essential to utilize preventive dental services to reduce dental pain in childhood.

## Data Availability

The datasets generated during and/or analyzed during the current study are available from the corresponding author on reasonable request.
